# MACRONUTRIENT BALANCE AND LONGEVITY: MECHANISTIC INSIGHTS OF DIETARY PROTEIN REGULATION ON AGING PHYSIOLOGY `MACRONUTRIENT BALANCE AND LONGEVITY: MECHANISTIC INSIGHTS OF DIETARY PROTEIN REGULATION ON AGING PHYSIOLOGY

**DOI:** 10.1093/geroni/igaf122.965

**Published:** 2025-12-31

**Authors:** Cristal Hill

**Affiliations:** University of Southern California, Los Angeles, California, United States

## Abstract

Neuroendocrine systems play a vital role in homeostatic processes that regulate growth, metabolism, and stress responsiveness, among others. In addition, dietary interventions such as CR or altering macronutrients are reported to modify neuroendocrine signaling to benefit health-span and lifespan. There is substantial evidence that reducing dietary protein intake (dietary protein restriction-DPR), without reducing caloric intake, improves glucose homeostasis, increases energy expenditure, and remodels white adipose tissue (WAT) by inducing thermoregulatory markers such as UCP1. Data from our lab shows that DPR robustly improves metabolic health via increased liver-derived FGF21 action in the brain and that DPR increases thermoregulatory genes, thus DPR represents a purely physiological model to study adipocyte biology. Specifically, the experiments in our lab are focused on identifying the potential mechanisms through which reducing protein intake alters cellular and molecular function in adipose tissue that impact metabolic health. The central focus of this work is to further connect the effects of protein intake on the impact of neuroendocrine signaling on aging physiology.

